# Meta-analysis of female stress urinary incontinence treatments with adjustable single-incision mini-slings and transobturator tension-free vaginal tape surgeries

**DOI:** 10.1186/s12894-015-0060-3

**Published:** 2015-07-07

**Authors:** Peng Zhang, Bohan Fan, Peng Zhang, Hu Han, Yue Xu, Biao Wang, Xiaodong Zhang

**Affiliations:** Urology department, Beijing Chaoyang hospital, Capital Medical University, 8 Gongren Tiyuchang NanluChaoyang District, Beijing, 100020 China; Urology department, Beijing Chaoyang hospital Capital Medical University, 8 Gongren Tiyuchang Nanlu, Chaoyang District Beijing, 100020 China; Urology department, Beijing Chaoyang hospital Capital Medical University, 8 Gongren Tiyuchang Nanlu,Chaoyang District, Beijing, 100020 China

**Keywords:** Single-incision mini-sling, Transobturator tension-free vaginal tape, Female stress urinary incontinence

## Abstract

**Background:**

The study on SIMS and SMUS as a whole by Alyaa Mostafa et al showed that after excluding the TVT-S sling, there is no significant difference in patient-reported cure rate and objective cure rate between these two methods. In this paper, we systematically evaluate the relevant data on SIMS-Ajust and TVT-O/TOT and further confirm their safety and effectiveness, providing reliable clinical evidence.

**Methods:**

By searching the Medline, Embase, Scopus, and Web of Science databases and the Cochrane Database of Systematic Reviews combined with manual searches, all reports on randomized controlled trials (RCTs) of single-incision mini-sling (SIMS-Ajust) and transobturator tension-free vaginal tape (TVT-O/TOT) surgeries were collected. Using RevMan 5.2 statistical software, the patient-reported cure rate, objective cure rate, operative time, postoperative pain, lower urinary tract injuries, groin pain, postoperative voiding difficulties, de novo urgency and/or worsening of preexisting surgery, vaginal tape erosion, repeated continence surgery, and other related data on both surgical methods were evaluated.

**Results:**

A total of 154 relevant research reports were retrieved, and five randomized controlled trials were included in this study, involving a total of 678 patients. The meta-analysis results show no significant difference in the patient-reported cure rate and objective cure rate between SIMS-Ajust and TVT-O/TOT [*RR* = 0.95, 95 % *CI* (0.87 to 1.04), *P* > 0.05; *RR* = 0.97, 95 % *CI* (0.90–1.05), *P* > 0.05]. With respect to operation time and groin pain, SIMS-Ajust outperforms TVT-O/TOT [MD = −1.61, 95 % CI (−2.48 to 0.74), *P* < 0.05; *RR* = 0.30, 95 % *CI* (0.11 to 0.85), *P* < 0.05]. In terms of postoperative pain, lower urinary tract injuries, postoperative voiding difficulties, de novo urgency and/or worsening of preexisting surgery, vaginal tape erosion, and repetition of continence surgery, there is no significant difference between SIMS-Ajust and TVT-O/TOT [*RR* = 0.50, 95 % *CI*(0.18–1.43), *P* > 0.05; *RR* = 2.82, 95 % *CI*(0.14–57.76), *P* > 0.05; *RR* = 0.64, 95 % *CI*(0.28–1.45), *P* > 0.05; *RR* = 1.06, 95 % *CI*(0.66–1.71), *P* > 0.05; *RR* = 1.04, 95 % *CI*(0.24–4.45), *P* > 0.05; *RR* = 1.64, 95 % *CI*(0.41–6.61), *P* > 0.05].

**Conclusions:**

SIMS-Ajust is safe and effective in the treatment of female stress urinary incontinence. Compared with TVT-O/TOT surgery, SIMS-Ajust surgery has the same high objective cure rate and patient-reported cure rate and low incidence of perioperative complications, in addition to its short operative time and low incidence of groin pain. Its long-term efficacy needs further observation.

## Background

The incidence rate of female stress urinary incontinence (SUI) in women in the United States is between 23 % and 67 % [1, 2]. Its risk factors include obesity and fertility, and studies have shown that when BMI increases by 5, the incidence rate of SUIrisk increases by 20 to 70 % [3]. Surgery has become a standard treatment for female stress urinary incontinence, and the surgical treatments can be roughly divided into six categories, namely, Marshall-Marchetti-Krantz operations (represented by the Burch operation), bladder neck suspension operations (represented by the Stamey and Raz operations), anterior vaginal wall repair operations, sling surgery, paraurethral injection, and artificial urinary sphincter [4]. As the operations are being improved and updated constantly, we are trying to find a treatment method that is not only effective, simple, easy to perform, with small trauma, and without long-term complications but also economical.

The SIMS-Ajust sling is a novel single-incision sling that appeared on the market in 2009 [5]. The patient-reported cure rate of SIMS-Ajust is between 73.9 % and 81.2 %, and its objective cure rate is between 76.8 % and 84.7 % [6–8]. The study on SIMS and SMUS as a whole by Alyaa Mostafa et al. [9] showed that after excluding the TVT-S sling, there is no significant difference in patient-reported cure rate and objective cure rate between these two methods. SIMS has an earlier and faster postoperative recovery. However, this report only performed an overall evaluation on single-incision mini-sling operations, including Mini-Arc, Ajust, Ophira, Needleless-Contasure, TFS, and Solyx, and did not include an individualized analysis on SIMS-Ajust. In particular, there is no report on the efficacy of SIMS-Ajust. Compared with the previous report, we have included two new randomized controlled trial literatures published in June and August 2013 [7, 10]. In this paper, we systematically evaluate the relevant data on SIMS-Ajust and TVT-O/TOT and further confirm their safety and effectiveness, providing reliable clinical evidence.

## Methods

### Data collection

Two urologists first extracted the relevant data and assessed their quality independently. The data were checked, and if there was a disagreement, experts were consulted to solve it. Computer searches were performed in the Medline, Embase, Scopus, and Web of Science databases and the Cochrane Database of Systematic Reviews. Manual searches were performed for meeting publications and abstracts of the International Continence Society (ICS), the International Urogynecological Association (IUGA), the American Urological Association (AUA), the European Association of Urology (EAU), and the Société Internationale d'Urologie (SIU). SIMS-Ajust and TVT-O/TOT randomized controlled trials (RCTs) were included. The key words stress urinary incontinence, single incision mini-sling, and Ajust were used for the searches. All the reports were published in English. The searched literature begins in 2009 and ends in August 2014. RCT literature quality was assessed using the Jadad score [11]: (1) whether it is a randomized controlled trial; (2) whether it is a blind test; and (3) any treatment for loss to follow-up and withdrawal.

Inclusion criteria: (1) RCTs of studies on the efficacy of surgeries for female stress urinary incontinence; (2) prospective studies; (3) trials of studies on the comparison of Ajust methods versus the TVT-O method or versus the TOT method; (4) the characteristic baseline of surgical objects is roughly the same; (5) observed indicators include the cure rate and perioperative complications; (6) with or without allocation concealment or with blind treatment. Exclusion criteria: (1) design is rigorous, but sample data and intervention means are not clear; (2) statistical method is not appropriate; (3) loss to follow-up rate is too high; (3) assessment criteria are not uniform, and therefore, the efficacy values cannot be combined.

### Statistical analysis

**Fig. 1:**
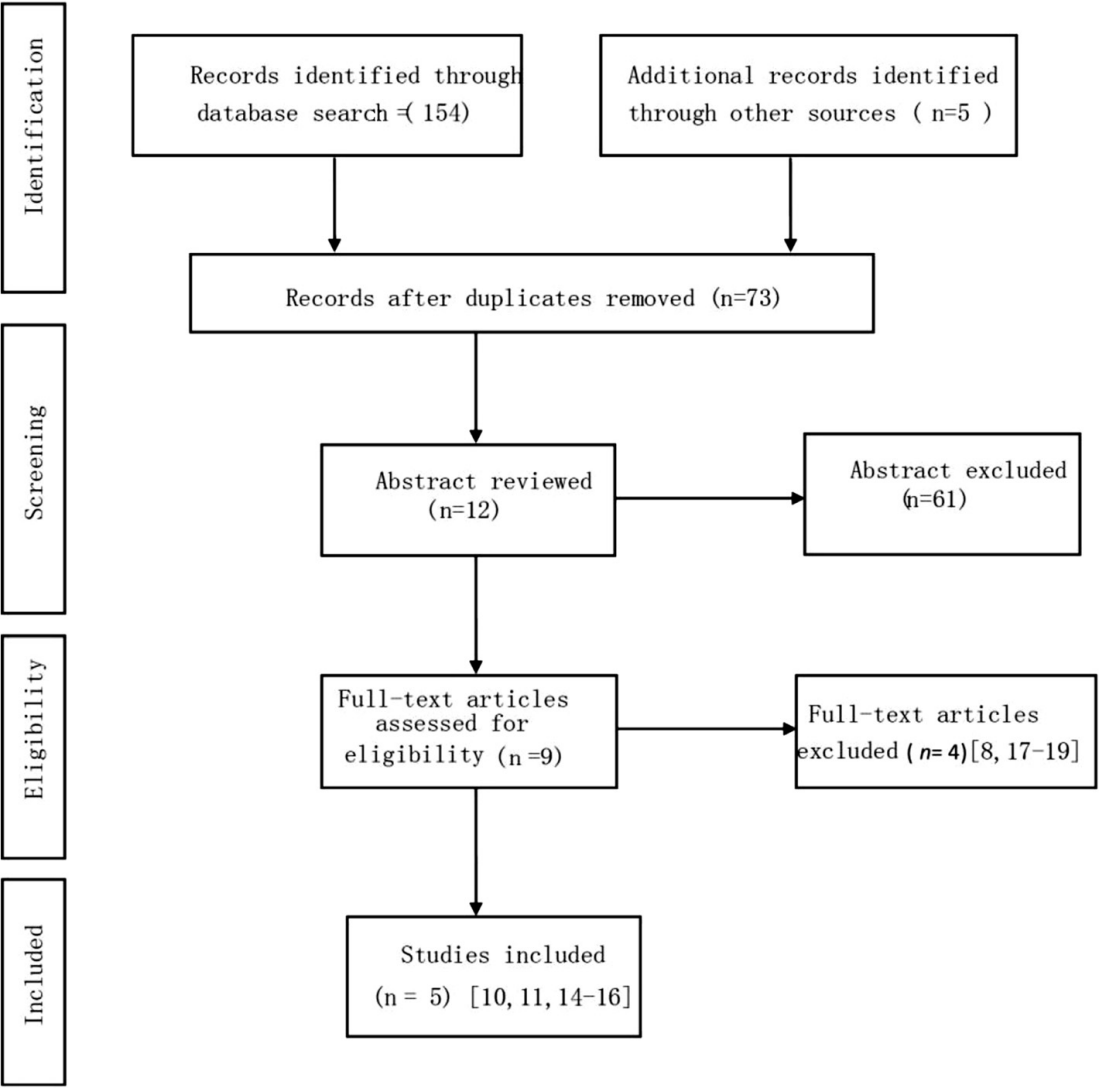
Preferred reporting items for systematic reviews and meta-analysis

RevMan v.5.2 (Cochrane Collaboration, Oxford UK) was used to perform meta-analysis on the included papers. Figure 1 shows the literature search process. Clinical examination and related urodynamic examination results were used to determine whether diagnosed female stress urinary incontinence patients had complications of overactive bladder, urge incontinence, and pelvic organ prolapse. We collected information about cure rate, operation time, and postoperative complications, including postoperative pain, lower urinary tract injuries, groin pain, postoperative voiding difficulties, de novo urgency and or worsening of preexisting surgery, vaginal tape erosion, and repeated continence surgery, and we analyzed the effectiveness and safety on this basis. The mean difference (MD) for quantitative data and relative risk (RR) for qualitative data were used as statistical values for efficacy analysis; the interval estimation used 95 % as the confidence interval [12]. The Q test was used to test the heterogeneity of the included studies. When the heterogeneity difference of each test had no statistical significance (*P* > 0.10, I^2^ < 50 %), a fixed effects model was used for meta-analysis; when the heterogeneity difference of a test had statistical significance (*P* < 0.10, I^2^ > 50 %), the reasons for heterogeneity were analyzed, and subgroup analysis was performed. A forest plot was generated with the aid of software; if the included number of studies was too low (*n* < 10), a funnel plot was not drawn [12].

## Results

A total of five RCT reports were selected, and the Jadad score for all five articles was three points (Table 1) [7,10,13,14,15] (SIMS-Ajust: *n* = 361, TVT-O/TOT: *n* = 317). A total of 13 people were lost to follow-up (SIMS-Ajust: *n* = 3, TVT-O/TOT: *n* = 10). The excluded papers and the reasons are listed in Table 2 [8,16-18].

**Table 1:** Jadad score

	Randomized controlled	Blinding method	Loss to follow-up/withdrawal	Total score
Dati 2012 [13]	2	0	1	3
Schweitzer 2012 [14]	2	0	1	3
Mostafa 2013 [10]	2	0	1	3
Grigoriadis 2013 [7]	2	0	1	3
Masata 2013 [15]	2	0	1	3

**Table 2:** Excluded literature

Boyers 2013 [8]	The same study as Mostafa 2013 [10]
Mostafa 2012 [16]	The same study as Mostafa 2013 [10]
Martan 2013 [17]	Two SIMS comparison randomized controlled trials
Palomba 2014 [18]	Three SIMS comparison randomized controlled trials

### Cure rate

**Fig. 2:**
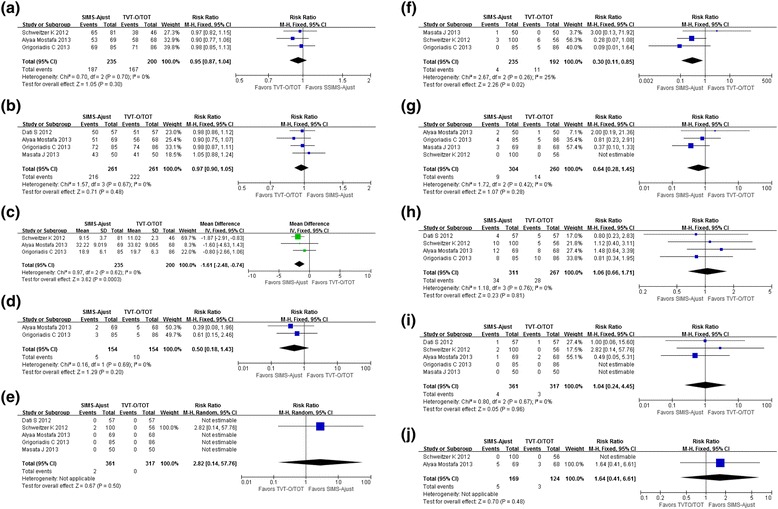
Meta-analysis results: (**a**) Patient-reported cure rate; (**b**) objective cure rate; (**c**) operative time; (**d**) postoperative pain; (**e**) lower urinary tract injuries; (**f**) groin pain; (**g**) postoperative voiding difficulties; (**h**) de novo urgency and/or worsening of preexisting surgery; (**i**) vaginal tape erosion; (**j**) repeat continence surgery. CI = confidence interval; M-H = Mantel-Haenszel; SIMS-Ajust = single-incision mini-sling Ajust

A total of three studies were included to compare the objective cure rate of two sling surgeries: there are 235 cases in the SIMS-Ajust group, and the number of objective cure cases is 187; there are 200 cases in the TVT-O/TOT group, and the number of objective cure cases is 167. Heterogeneity test I^2^ = 0 %, *P* > 0.1, and therefore, the included literature can be considered homogeneous, and a fixed model is used for the statistical analysis. The results show that the objective cure rate of the two groups has no significant difference [*RR* = 0.95, 95 % CI (0.87 to 1.04), *P* > 0.05] (see Fig. 2a). A total of four studies were included for the comparison of the patient-reported cure rate: 261 cases in the SIMS-Ajust group with 216 patient-reported cure cases; 261 cases in the TVT-O/TOT group with 222 patient-reported cure cases. The heterogeneity test I^2^ = 0 %, *P* > 0.1, and therefore, the included reports can be considered homogeneous. A fixed model is then used for the statistical analysis, and the results show no significant difference in the patient-reported cure rate in the treatment of female stress urinary incontinence between SIMS-Ajust and TVT-O/TOT [*RR* = 0.97, 95 % CI (0.90 to 1.05), *P* > 0.05] (see Fig. 2b).

### Surgical Information

A total of three studies were used for the statistical analysis of operation time, with 235 cases in the SIMS-Ajust group and 200 cases in the TVT-O/TOT group. Heterogeneity test I^2^ = 0 %, *P* > 0.1; therefore, the included reports can be considered homogeneous, and the analysis results show that SIMS-Ajust has a shorter operation time than TVT-O/TOT in the treatment of female stress urinary incontinence [WMD = −1.61 min, 95 % CI (−2.48 to −0.88), *P* < 0.05] (see Fig. 2c). A total of two studies were used for the statistical analysis of postoperative pain: 154 cases are in the SIMS-Ajust group with five cases having postoperative pain, and 154 cases are in the TVT-O/TOT group with 10 cases having postoperative pain. Heterogeneity test I^2^ = 0 %, *P* > 0.1, and therefore, the included reports can be considered homogeneous, and a fixed model is used for the statistical analysis. The results show that there is no significant difference in the incidence rate of postoperative pain between SIMS-Ajust and TVT-O/TOT [*RR* = 0.50, 95 % CI (0.18 to 1.43), *P* > 0.05] (see Fig. 2d).

### Complications

The statistics on five postoperative complications show that the incidence rate of postoperative groin pain by SIMS-Ajust is significantly less than for TVT-O/TOT [*RR* = 0.30, 95 % CI (0.11 to 0.85), <0.05]. The statistics on lower urinary tract injuries, postoperative voiding difficulties, de novo urgency and/or worsening of preexisting surgery, and vaginal tape erosion show no significant difference between the two operations [*RR* = 0.50, 95 % CI (0.18 to 1.43), *P* > 0.05; *RR* = 2.82, 95 % CI (0.14 to 57.76), *P* > 0.05; *RR* = 0.64, 95 % CI (0.28 to 1.45), *P* > 0.05; *RR* = 1.06, 95 % CI (0.66 to 1.71), *P* > 0.05; *RR* = 1.04, 95 % CI (0.24 to 4.45), *P* > 0.05] (see Fig. 2e-i).

### Repeat of continence surgery

In total, three studies performed statistical analysis on repeated continence surgery. In the 169-case SIMS-Ajust group, five cases had repeated continence surgery; in the 124-case SMUS group, three cases required repeated continence surgery. Heterogeneity test I^2^ = 0 %, *P* > 0.1, so the included reports can be considered homogeneous. A fixed model is used for statistical analysis, and the results showed no significant difference in the incidence rate of repeated continence surgery between these two groups [*RR* = 1.64, 95 % CI (0.41 to 6.61), *P* > 0.05] (Fig. 2j).

### Publication bias

A total of five (<10) RCTs were included in this study, so funnel plot analysis was not performed to detect publication bias.

## Discussion

A tension-free midurethral sling is a grade A recommendation according to the guide for stress urinary incontinence treatment [19]. Midurethral slings can be divided into three generations [20]: In 1995, Ulmsten and Petros [21] established retropubic tension-free vaginal tape (TVT) as the first generation of sling operations to treat urinary incontinence, and it was soon widely accepted and considered the standard operation for the treatment of female stress urinary incontinence (SUI). Although this operation has effective results, bladder perforation and other complications [22] prompted people to continue to search for other sling methods. The second generation of sling operations for the treatment of urinary incontinence is the transobturator TVT-O and TOT operation, and meta-analysis [23] showed no significant difference in efficacy between retropubic vaginal tape operation and transobturator operation. Although this operation avoids bladder perforation, postoperative thigh pain or groin pain becomes a common complication, and its incidence rate is between 1.6 % and 8.2 % [24,25]. Various vaginal single-incision midurethral sling operations are the third generation of urinary incontinence operations [20]. Compared with traditional operations, single-incision vaginal sling operations 1) have a shorter sling, with less foreign material being inserted into human body, thereby reducing the adverse reactions to foreign material; 2) have less injury to the patient, thereby reducing possible perforation infections; 3) avoid having bladder, obturator nerves, and blood vessels in the puncture path, which is therefore safer than the traditional retropubic midurethral sling (RP-TVT) and TVT-O/TOT.

The Ajust sling is one of the single-incision vaginal slings that appeared on the market in 2009 [5]. Its puncture method is to use a specially designed anchor to fix the sling on the obturator membrane without letting both ends penetrate through the skin. After implantation, the tightness of the sling is adjusted through the device. In comparison, a TVT-O/TOT sling penetrates through the inner side of the thigh. Therefore, anatomically, it seems that SIMS-Ajust might have a lower cure rate or increased cases of repeat continence surgery due to its weaker anchor force. However, in reality, SIMS-Ajust has turned this factor entirely into an advantage. Our meta-analysis of the two tapes showed that SIMS-Ajust had enough anchor force in practice and with low groin pain. After comparing the five studies included in this meta-analysis, we found no significant difference in the patient-reported cure rate and objective cure rate between SIMS-Ajust and TVT-O/TOT. In addition, there is no significant difference in the comparison of the incidence rate of repeat continence surgery between SIMS-Ajust and TVT-O/TOT. The follow-up periods of both the reports included in this study are longer than 12 months. It is reasonable to say that the mid-term efficacy of SIMS-Ajust is reliable. This result indicates that although the SIMS-Ajust puncture path is short, its anchor has enough force to fix the sling in the midurethral position and cure stress urinary incontinence. In a 90-sample study by Mohamed Abdel-Fattah et al. [6], there were two cases of less effective slings that failed when removed, which corroborates the reliability of the anchor force from the side. The effectiveness of SIMS-Ajust is similar to the effectiveness of traditional TVT-O/TOT.

Similar to other single incision slings, the Ajust puncture does not require an incision on the inner side of the thigh or suprapubic, which reduces the risks of blood vessel, nerve, and visceral injuries. There are no cases of blood vessel or nerve injuries and other serious complications in the studies included in this meta-analysis. SIMS-Ajust has a low postoperative groin pain incidence rate. In the RCT study of Grigoriadis C. [7], SIMS-Ajust has no cases of postoperative groin pain, while TVT-O/TOT has five cases of postoperative groin pain, which disappeared 15 days after the operation. Therefore, in terms of the appearance of groin pain, the advantage of SIMS-Ajust is obvious. The included studies show that SIMS-Ajust has a shorter operation time than SMUS by 1–3 min, indicating that its operation is simpler and more convenient, this shorter may have no contribution to improvement of the safety of the operation. In the comparison of other complications, postoperative pain, lower urinary tract injuries, postoperative voiding difficulties, de novo urgency and/or worsening of preexisting surgery, and vaginal tape erosion are similar for both operation methods. Therefore, we can say that SIMS-Ajust is a safe operation for treating female stress urinary incontinence. Meanwhile, Dwayne Boyers et al. [8] performed statistical analysis on the health services and patient quality adjusted life-years (QALYs) of the same group of people in Alyaa Mostafa’s study to assess the health costs, and the results showed that because SIMS-Ajust is performed under local anesthesia, the cost is reduced according to one-year follow-up cost-effectiveness.

There are limitations of this study: (1) the number of included RCT studies is small (5), and none of them are double-blind studies; (2) observation indicators and assessment methods are different, resulting in the loss of some study data; (3) the 95 % confidence interval of some observation indicators is too wide, which requires more studies; and (4) possible gray literature may exist and lead to publication bias.

## Conclusions

In summary, SIMS-Ajust surgical treatment for female stress urinary incontinence is safe and effective. SIMS-Ajust surgery, compared with TVT-O/TOT surgery, has the same high patient-reported and objective cure rates and low perioperative complications incidence rate. In addition, it has a short operation time and a low incidence rate of groin pain. However, as some of the studies included in this meta-analysis have a short follow up time, and meta-analysis requires continuous updates, the long-term efficacy needs further observation.

## Consent

Written informed consent was obtained from the patient’s guardian/parent/next of kin for the publication of this report and any accompanying images.

## Abbreviations

SIMS-Ajust: Adjustable single-incision mini-sling
TVT-O/TOT: Transobturator tension-free vaginal tape
RCTs: Randomized controlled trials
SUI: Stress urinary incontinence
SMUS: Standard midurethral slings

## References

[1] Fultz NH, Herzog AR (2000). Prevalence of urinary incontinence in middle aged and older women: a survey based methodological experiment. J Aging Health.

[2] Novielli K, Simpson Z, Hua G, Diamond JJ, Sultana C, Paynter N. Urinary incontinence in primary care: a comparison of older African-american and Caucasian women. Int Urol Nephrol 2003;35(3):423–8. 10.1023/b:urol.0000022868.73066.9a 15160551

[3] Osborn DJ, Strain M, Gomelsky A, Rothschild J, Dmochowski R. Obesity and female stress urinary incontinence. Urology 2013;82(4):759–63. 10.1016/j.urology.2013.06.020 23972338

[4] Thüroff JW, Abrams P, Andersson KE, Artibani W, Chapple CR, Drake MJ, et al. EAU Guidelines on Urinary Incontinence. Actas Urol Esp. 2011;35(7):373–88. 10.1016/j.acuro.2011.03.012 21600674

[5] Kennelly MJ, Myers EM (2011). Retropubic and Transobturator Slings: Still Useful or Should All Patients Be Treated with Mini-slings?. Curr Urol Rep.

[6] Abdel-Fattah M, Agur W, Abdel-All M, Guerrero K, Allam M, Mackintosh A, et al. Prospective multi-centre study of adjustable single-incision mini-sling (Ajust ® ) in the management of stress urinary incontinence in women: 1-year follow-up study. BJUI 2011;109:880–6. 10.1111/j.1464-410X.2011.10471.x 21883844

[7] Grigoriadis C, Bakas P, Derpapas A, Creatsa M, Liapis A. Tension-free vaginal tape obturator versus Ajust adjustable single incision sling procedure in women with urodynamic stress urinary incontinence. Eur J Obstet Gynecol Reprod Biol. 2013;170(2):563–6. 10.1016/j.ejogrb.2013.07.041 23972452

[8] Boyers D, Kilonzo M, Mostafa A, Abdel-Fattah M. Comparison of an adjustable anchored single-incision mini-sling, Ajust®, with a standard mid-urethral sling, TVT-OTM: a health economic evaluation. BJU Int, 2013;112:1169–77. 10.1111/bju.12388 24053310

[9] Mostafa A, Lim CP, Hopper L, Madhuvrata P, Abdel-Fattah M (2014). Single-Incision Mini-Slings Versus Standard Midurethral Slings in Surgical Management of Female Stress Urinary Incontinence: An Updated Systematic Review and Meta-analysis of Effectiveness and Complications. Eur Urol.

[10] Mostafa A, Agur W, Abdel-All M, Guerrero K, Lim C, Allam M, et al. Multicenter prospective randomized study of single-incision mini-sling vs tension-free vaginal tape-obturator inmanagement of female stress urinary incontinence: a minimum of 1-year follow-up[J]. Urology. 2013;82(3):552–9. 10.1016/j.urology.2013.02.080 23845666

[11] Jadad AR, Rennie D. The randomized controlled trial gets a middleaged checkup. JAMA 1998;279:319–20. 10.1001/jama.279.4.319 9450719

[12] Higgins JPT, Green S, editors. Cochrane Handbook for Systematic Reviews of Interventions, v.5.1.0. http://www.cochrane.org/. Accessed October 14, 2012.

[13] Dati S, Rombola P, Cappello S, Piccione E. Single-incision minisling (AJUST) vs obturator tension-free vaginal shortened tape (TVTABBREVO) in surgical management of female stress urinary incontinence. Int J Gynecol Obstet 2012;119:S670.

[14] Schweitzer KJ, Cromheecke GJ, A. L. Milani, H. W. Van Eijndhoven, D. Gietelink 4, E. Hallenleben, et al. A randomized controlled trial omparing the TVT-O® with the Ajust® as primary surgical treatment of female stress urinary incontinence[J]. Int Urogynecol J. 2012;23(2):S77–78.

[15] J. Masata, K. Svabik, P. Hubka, R. Elhaddad, A. Martan. Comparison of the safety and peri-operative complications of transobturator introduced tension-free vaginal tape (TVT-O) and single-incision tape with adjustable length and anchoring mechanism (Ajust) in a randomized trial: short term results[J]. Int Urogynecol J. 2013;24(1):S114–5.

[16] Mostafa A, Agur W, Abdel-All M, Guerrero K, Lim C, Allam M, et al. A multicentre prospective randomised study of single-incision mini-sling (Ajust®) versus tension-free vaginal tape-obturator (TVT-O™) in the management of female stress urinary incontinence: pain profile and short-term outcomes. Eur J Obstet Gynecol Reprod Biol. 2012 ,165(1):115–21. 10.1016/j.ejogrb.2012.06.022 22917936

[17] Alois M, Jan K, Jaromir M, Kamil S, Michael H A,Lukas H, et al. Prospective Randomized Study of MiniArc and Ajust Single Incision Sling Procedures. LUTS, 2013;6(3):172–4. 10.1111/luts.12041 26663600

[18] Palomba S, Falbo A, Oppedisano R, Torella M, Materazzo C, Maiorana A, et al. A randomized controlled trial comparing three single-incision minislings for stress urinary incontinence. Int Urogynecol J. 2014;25(10):1333–41. 10.1007/s00192-014-2383-0 24737301

[19] Lucas MG, Bosch RJ, Burkhard FC, Cruz F, Madden TB, Nambiar AK, et al. EAU guidelines on surgical treatment of urinary incontinence. Actas Urol Esp. 2013;37(8):459–72. 10.1016/j.acuro.2013.02.002 23835037

[20] Maslow K, Gupta C, Klippenstein P, Girouard L. Randomized clinical trial comparing TVT Secur system and trans vaginal obturator tape for the surgical management of stress urinary incontinence. Int Urogynecol J 2014;25:909–14. 10.1007/s00192-013-2312-7 24452619

[21] Ulmsten U, Petros P. Intravaginal slingplasty (IVS):an ambulatory surgical procedure for treatment of female urinary incontinence. Scand J Urol Nephrol, 1995;29:75–82. 10.3109/00365599509180543 7618052

[22] Deng DY, Rutman M, Raz S, Rodriguez LV. Presentation and management of major complications of midurethral slings: Are complications under-reported? Neurourol Urodyn 2007;26:46–52. 10.1002/nau.20357 17149713

[23] Novara G, ArtibaniVW, Barber MD, Chapple CR, Costantini E, Ficarra V, et al. Updated Systematic Review and Meta-Analysis of the Comparative Data on Colposuspensions, Pubovaginal Slings, and Midurethral Tapes in the Surgical Treatment of Female Stress Urinary Incontinence. Eur Urol, 2010;58(2):218–38. 10.1016/j.eururo.2010.04.022 20434257

[24] Bianchi-Ferraro AM, Jarmy-Dibella ZI, de Aquino Castro R, Bortolini MA, Sartori MG, Girão MJ. Randomized controlled trial comparing TVT-O and TVT-S for the treatment of stress urinary incontinence: 2-year results. Int Urogynecol J, 2014;25(10):1343–8. 10.1007/s00192-014-2352-7 24643378

[25] Tincello DG, Botha T, Grier D, Jones P, Subramanian D, Urquhart C, et al. The TVT Worldwide Observational Registry for Long-Term Data: safety and efficacy of suburethral sling insertion approaches for stress urinary incontinence in women. J Urol, 2011;186(6):2310–5. 10.1016/j.juro.2011.07.078 22014817

